# Deep Ensemble Learning Based on Multi-Form Fusion in Gearbox Fault Recognition

**DOI:** 10.3390/s25164993

**Published:** 2025-08-12

**Authors:** Xianghui Meng, Qingfeng Wang, Chunbao Shi, Qiang Zeng, Yongxiang Zhang, Wanhao Zhang, Yinjun Wang

**Affiliations:** 1 State Key Laboratory of Coal Mine Disaster Prevention and Control, CCTEG, Chongqing Research Institute, Chongqing 400039, China; 2 State Key Laboratory of Mechanical Transmission for Advanced Equipment, Chongqing University, Chongqing 400044, China; 3 China North Vehicle Research Institute, Beijing 100072, China; 4 Chongqing Key Laboratory of Green Design and Manufacturing of Intelligent Equipment, Chongqing Technology and Business University, Chongqing 400067, China

**Keywords:** ensemble learning, multi-information fusion, fault identification, gearbox

## Abstract

Considering the problems of having insufficient fault identification from single information sources in actual industrial environments, and different information sensitivity in multi-information source data, and different sensitivity of artificial feature extraction, which can lead to difficulties of effective fusion of equipment information, insufficient state representation ability, low fault identification accuracy, and poor robustness, a multi-information fusion fault identification network model based on deep ensemble learning is proposed. The network is composed of multiple sub-feature extraction units and feature fusion units. Firstly, the fault feature mapping information of each information source is extracted and stored in different sub-models, and then, the features of each sub-model are fused by the feature fusion unit. Finally, the fault recognition results are obtained. The effectiveness of the proposed method is evaluated by using two gearbox datasets. Compared with the method of simple stacking fusion and single measuring point without fusion, the accuracy of each type of fault recognition of the proposed method is close to 100%. The results show that the proposed method is feasible and effective in the application of gearbox fault recognition.

## 1. Introduction

Gearbox plays an important role in rail transit, national defense, aerospace, manufacturing, and other fields [1,2,3]. As the most widely used transmission system in mechanical equipment, the gear transmission system has excellent characteristics such as compact structure, stable operation, high efficiency, and long service life. It is widely used in the transmission system of various heavy machinery [4,5,6]. Gearboxes often work in complex and harsh environments, so they are prone to failure during operation, resulting in downtime [7], and even serious economic and safety losses. Therefore, the research on condition monitoring and the fault identification method of gearbox is of great significance for modern industrial production [8,9].

Considering the problems of the inaccurate acquisition of gearbox fault information and poor fault identification effects in the actual industrial environment, many scholars have conducted extensive and in-depth research on gearbox fault diagnosis and recognition technology, and achieved remarkable results. Deep learning algorithms like Convolutional Neural Networks (CNNs) and Recurrent Neural Networks (RNNs) employ hierarchical neural networks to automatically learn and represent data features, eliminating manual efforts required by traditional feature extraction methods while excelling at processing large-scale complex datasets, which has enabled their widespread applications in data mining, fault diagnosis, and related domains. Wang et al. [10] developed a deep learning framework for gearbox condition monitoring and life prediction using a deep neural network (DNN), and achieved good results. Xu et al. [11] proposed a deep transfer convolutional neural network (TCNN) fault diagnosis method. Duan et al. [12] proposed a deep focus parallel convolutional neural network (DFPCN) fault recognition method for the limitations of unbalanced data samples in deep learning, which successfully improved the accuracy. Peng et al. [13] divided the vibration signal into multiple signal components, and proposed a multi-branch multi-scale convolutional neural network from multiple time scales to learn and fuse rich complementary fault information. Qiao et al. [14] combined a convolutional neural network and a long short-term memory (LSTM) neural network to propose a dual-input model, which solved the problem of equipment fault identification under different working conditions. In terms of network interpretability, Chen and Enrico et al. [15,16] have conducted a comprehensive review and related research.

However, most of the above studies use single information source data for fault diagnosis. However, in the actual industrial scene, the internal structure of the gearbox is complex, the internal space is compact, and the faults are diverse. The location and number of sensors are limited by the ability of feature representation and the accuracy of perception. The data value density of a single information source is low, and the fault information is insufficient, which makes the fault difficult to identify. Even once the only sensor fails, it may cause false identification. At the same time, multiple information sources also have problems such as different information sensitivities and uneven fault feature representation capabilities. Therefore, some scholars have attempted to use information fusion technology to fuse multi-information source data to further improve the ability of fault identification. In general, multi-information fusion can be divided into three levels: data-level fusion, feature-level fusion, and decision-level fusion.

Data-level fusion is to fuse the original observation data, which can achieve high-precision performance. Considering the time and space information of the original data from multiple vibration sensors, Xia et al. [17] proposed a fault diagnosis method for rotating machinery based on a convolutional neural network. On the basis of a local filtering algorithm, Tian et al. [18] proposed a distributed fusion filter by using matrix a weighted fusion estimation algorithm, which is used for distributed fusion of multi-sensor data. Shao et al. [19] used the fusion strategy of a stacked wavelet autoencoder and weighted allocation for multi-sensor data fusion, and performed fault identification on the fused planetary gearbox data. Unfortunately, although the data-level fusion has a small loss of information, the amount of data to be processed is huge, and the original data are susceptible to noise pollution, which can easily lead to performance degradation.

Feature-level fusion generally combines multiple feature vectors into one or more vectors through certain processing. Compared with data-level fusion, feature-level fusion has a certain compression of the data, which shows the characteristics of the data in a more refined way and facilitates real-time processing. Xie et al. [20] proposed a new intelligent diagnosis method based on multi-sensor fusion (MSF) and a convolutional neural network. The method based on Principal Component Analysis (PCA) is used to fuse multi-signal data into three-channel RGB images. Ye et al. [21] proposed a multi-channel fault diagnosis data fusion method based on deep belief network (DBN) and random forest fusion, which fused multiple statistical features obtained from a time domain and a frequency domain to improve the fault diagnosis accuracy of the main reducer. Qian et al. [22] proposed a motor fault diagnosis method based on multi-feature fusion of a convolutional neural network. The signal is input into multi-time windows synchronously, which can identify motor faults with high accuracy. Liu et al. [23] proposed a fault diagnosis model based on multi-dimensional feature fusion and ensemble learning, as well as the optimal feature subset obtained by principal component analysis and feature fusion.

As for decision-level fusion, it is the final decision that integrates the decisions of various information sources. The final decision is mostly completed by voting mechanism, D-S theory and other technologies. Niu et al. [24] trained multiple classifiers for multiple sensor data sources, and used correlation analysis to perform decision-level fusion on the recognition results of multiple classifiers, which improved the accuracy of fault recognition. Mihajlo et al. [25] also constructed multiple SVM models, and used the maximum entropy principle to fuse the results of multiple models at the decision level to identify decentralized faults. Xiong et al. [26] constructed multiple KNN models, and used a static discount factor to optimize the D-S algorithm, and fused the results of different models at the decision level, which can eliminate some interference factors in fault identification. Huo et al. [27] proposed a fault diagnosis framework for rotating machinery based on multi-sensor data by combining thermal imaging with vibration measurements. The weighted D-S evidence theory is used to fuse at the decision level. Decision-level fusion can fuse different types of sensors and has good fault tolerance, but the loss of original information is large, which may lead to the loss of important fault information and the weakening of fault information representation ability. At the same time, the requirement of prior knowledge acquisition is also high.

Although some efforts have been made to solve the problem of multi-information source fusion in mechanical fault diagnosis, most of them do not take into account the correlation between various information sources, and lose more information, resulting in unsatisfactory fusion effect. Ensemble learning completes the corresponding task by constructing and combining multiple base learners to obtain a new strong learner from multiple base learners. Ensemble learning can decrease the difference between base learners, reduce the overall error rate, and improve the generalization ability of the model. Based on the above, a multi-information fusion fault identification network model based on deep ensemble learning is proposed in this study. The network model consists of a multi-information source fault feature extraction unit, a feature fusion unit and an output layer. The input of the network is multiple one-dimensional vibration time-domain signals, which belongs to the end-to-end network form, reduces the influence of manually selecting fault features on network performance, and retains the original fault information to the greatest extent. The multi-source fault feature extraction unit extracts and stores the fault feature mapping information of each information source. The feature fusion unit fuses the fault feature mapping information of each information source into the overall fault feature matching information at the feature level, and finally, obtains the final fault identification result through the decision output layer.

The main contributions of our work can be summarized as follows.

(1) The paper proposes a hierarchical neural framework integrating parallel 1D-CNN subnets and adaptive feature fusion mechanisms to address multi-source information sensitivity discrepancies;
(2) Develops a specialized CNN architecture optimized for temporal vibration pattern extraction through dilated convolutions and progressive feature abstraction;
(3) Establishes a data fusion methodology bridging ensemble learning theory with multi-sensor feature integration.

The rest of this study is recognized as follows. Section 2 introduces the basic theory of CNN and ensemble learning. Section 3 proposes a multi-information fusion fault identification model structure based on deep ensemble learning and introduces it in detail. Section 4 uses two gearbox datasets to verify the effectiveness of the proposed method. The conclusion is drawn in Section 5.

## 2. Theory Background

### 2.1. Multi-Information Fusion Based on Ensemble Learning

**Figure 1:**
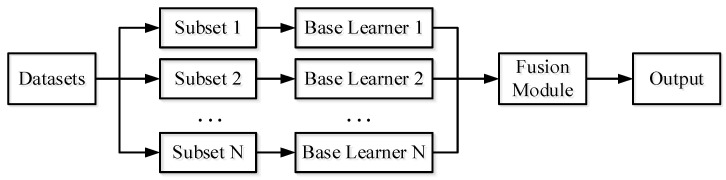
Ensemble learning.

Ensemble learning completes the corresponding task by constructing and combining multiple learners to obtain a strong learner from multiple weak base learners. Ensemble learning can be considered as a process of everyone picking up firewood, and the key is the construction and fusion of base learners. According to the different selection of base learners and fusion methods, ensemble learning can be divided into stacking [28], bagging [29], and boosting [30]. The general process of ensemble learning is shown in Figure 1. Ensemble learning generally divides the dataset into multiple data subsets by self-service sampling or data processing, and then trains different base learners for different data subsets. Finally, it is merged into a strong learner by fusion methods such as voting.

**Figure 2:**
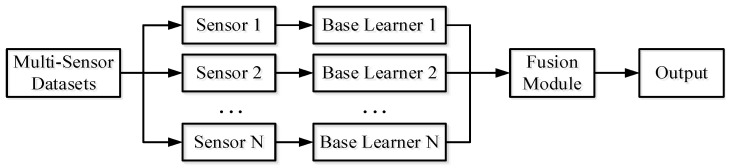
Multi-information fusion.

Information fusion is a multi-level and multi-level data processing process, which mainly completes the automatic detection, correlation, correlation, estimation, and combination of data from multiple information sources. It can be seen that there are conceptual similarities between data fusion and ensemble learning. Therefore, this paper uses the concept of ensemble learning to perform data fusion. Referring to the schematic diagram of ensemble learning in Figure 1, the proposed data fusion schematic process is shown in Figure 2. It is to divide the collected multi-information source dataset into various datasets according to different sources, and then train different base learners for different information source data. Finally, it is fused into a strong learner by fusion methods such as the voting method, and finally the result of data fusion can be obtained.

### 2.2. Convolutional Neural Network

**Figure 3:**
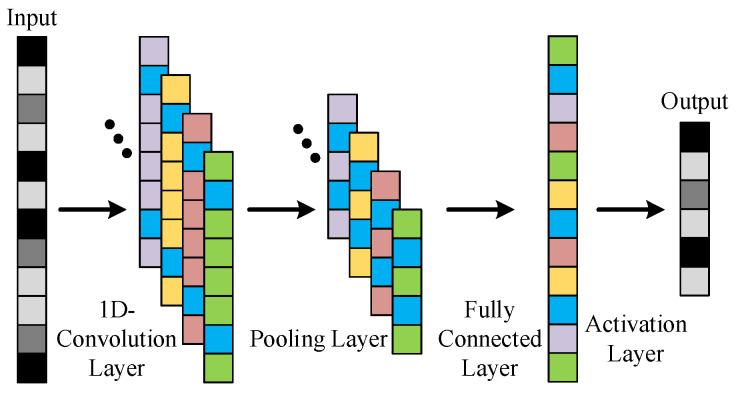
Structure of 1D-CNN.

Different from the two-dimensional convolutional network in the field of image recognition, the one-dimensional convolutional neural network (1D-CNN) is more widely used in the field of mechanical fault recognition. Its basic structure is shown in Figure 3, which consists of input, one-dimensional convolutional layer, pooling layer, fully connected layer, and output layer. The input in the field of mechanical fault identification is usually the time domain signal collected by the condition monitoring system.

The convolutional layer is the core part of the one-dimensional convolutional neural network. The idea of weight sharing reduces the number of network parameters, reduces training difficulties, improves efficiency, and reduces the risk of network overfitting. Weight sharing is the convolution operation with the same convolution kernel and input, so the weight is the same, that is, sharing. The calculation formula of one-dimensional convolution operation is

(1) $$if\frac{l-k}{s}\in N^{*},y_{i}=\sum_{j=1}^{k}w_{j}\cdot x_{(i-1)s+j}+b_{i},i\in [1,(l-k)/s+1]$$

(2) $$if\frac{l-k}{s}\notin N^{*},\left\{\begin{array}{l} y_{i}=\sum_{j=1}^{k}w_{j}\cdot x_{(i-1)s+j}+b_{i},i\in [1,\left\lfloor (l-k)/s+1\right\rfloor ]\\ y_{i}=\sum_{j=1}^{l-i\times s}w_{j}\cdot x_{i\times s+j}+b_{i},i\in \left\lceil (l-k)/s\right\rceil \end{array}\right.$$

where *l* is the length of the input one-dimensional original vibration signal *x*, *w* is the convolution kernel, *b* is the offset, *k* is the length of *w*, *s* is the convolution step length. N* is the set of positive integers, and ⌊⋅⌋ and ⌈⋅⌉ denote the downward and upward integers, respectively. *y_i_* is the *i* th element of the output after convolution operation.

## 3. Deep Ensemble Learning for Multi-Information Fusion

### 3.1. Deep Ensemble Learning Architecture

**Figure 4:**
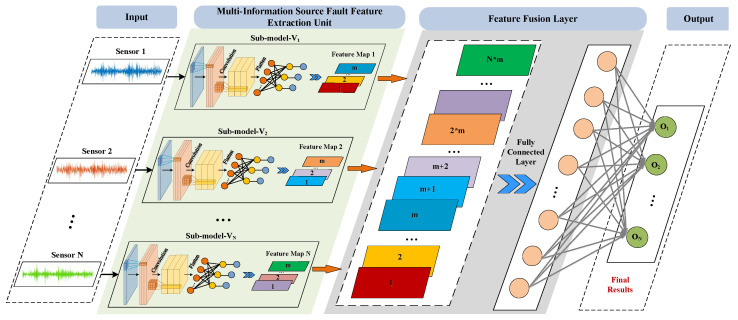
The flow diagram of the proposed multi-information fusion network architecture.

Revised version with technical enhancement: Drawing upon the principles of ensemble learning, we construct specialized diagnostic modules tailored to heterogeneous data streams. Each domain-specific classifier undergoes localized training at its native data generation node, where it demonstrates superior diagnostic performance for localized data patterns. Through an adaptive feature fusion mechanism, these distributed models collaboratively extract distinctive feature representations while preserving source-specific attributes. The cross-source correlation analysis is achieved via a hierarchical neural architecture that integrates both parallel feature channels and vertical decision layers, as detailed in our proposed framework (Figure 4). This modular design enables dynamic knowledge transfer between decentralized data sources while maintaining domain adaptation capabilities.

The multi-information fusion deep neural network is composed of a multi-information source fault feature extraction unit, a feature fusion unit, and an output layer. The input of the multi-information fusion deep neural network is multiple one-dimensional vibration time-domain signals. The input of the network does not require manual processing and extraction of fault signal features. It belongs to the end-to-end network form, which reduces the influence of manually selecting sample fault features on network performance. The multi-information source fault feature extraction unit is composed of multiple CNN fault feature extraction sub-models. The structure of each sub-model is the same, and the fault feature mapping information of each information source is extracted and stored, respectively. The feature fusion unit is used to fuse the features extracted from different information source models. The fault feature mapping information of each information source is fused into the overall fault feature mapping information, and finally, the recognition result of the entire network is obtained through the decision output layer.

### 3.2. Multi-Information Source Fault Feature Extraction Unit

The multi-modal data integration strategy developed in this research operates within hierarchical feature representations, necessitating the preliminary isolation of defect-characteristic correlations across heterogeneous data streams as its foundational phase. This methodology deploys structurally homogeneous CNN-based feature extractors tailored for individualized input modalities (*V*_1,_ *V*_2_, …, *V_N_*), where *N* denotes the quantity of input sources, through parallelized convolutional architectures specifically optimized for multi-source pattern recognition. These dedicated feature isolation modules implement a Waveform-Dilated Convolutional Neural Network (WDCNN) framework, whose detailed architectural configuration—including dilated convolution layers and adaptive pooling mechanisms is systematically organized in Table 1 to demonstrate parameter hierarchies and kernel dimension progression across network depths. Each independently trained channel-specific sub-network (*V*_1_ to *V_N_* pathways) focuses on distilling vibration spectrum fingerprints through depth wise separable convolutions while maintaining weight-sharing constraints in fully connected layers for cross-modal comparability. Rectified Linear Units (ReLU) are employed to introduce nonlinearity, while the final fully connected layer utilizes the Softmax activation function for fault classification. The pooling layers adopt unparameterized max-pooling operations, and no activation is applied post-pooling.

During the fault identification phase, multi-source data collected from diverse information channels are processed through a specialized fault feature extraction framework. All raw vibration signals underwent z-score normalization (0, 1) prior to network input, ensuring consistent feature scaling across heterogeneous sensor channels. Individual feature extractors within this framework are selectively activated based on their assigned data streams, enabling the parallel extraction of distinct fault signatures from heterogeneous sources. The architecture employs a modular design where the quantity of extractors corresponds precisely to the number of input channels, ensuring dedicated analysis of each data type for targeted fault characterization. Each optimized extraction module, configured as a sequential stack comprising a 1D convolutional layer, max-pooling operation, and dense connection layer, focuses on uncovering source-specific diagnostic patterns. The resultant output from the *i*-th module mathematically represents the transformed feature space derived from its associated information channel, facilitating subsequent classification tasks through hierarchical pattern mining across multiple data dimensions. The output of the *i*-th sub-network unit is defined as:

(3) $$S^{i}=f_{\varphi_{i}}^{i}(x)=[s_{1}^{i},s_{2}^{i},\ldots ,s_{m}^{i}]$$

where *m* denotes the dimensionality of the latent feature vector generated by individual extraction modules, and *φ_i_* represents the trainable parametric weights associated with the *i*-th computational sub-unit. The synthesized output of the integrated multi-source diagnostic framework is formulated through the hierarchical fusion of these modular components, mathematically expressed as the weighted aggregation of all subsystem-derived feature manifolds. This composite representation strategically preserves both the source-specific discriminative patterns extracted by parallel channels and the cross-modal correlations learned through adaptive parameterization, thereby establishing an enriched basis for downstream fault classification. The architecture’s modular design ensures mathematical tractability while maintaining structural flexibility to accommodate heterogeneous sensor inputs through its dimensionally consistent transformation schema.

(4) $$S=[S^{1},S^{2},\;\ldots \;,S^{n}]=\left[\begin{array}{cccc} s_{11} & s_{12} & \ldots & s_{1n}\\ s_{21} & s_{22} & \ldots & s_{2n}\\ \ldots & \ldots & \ldots & \ldots \\ s_{m1} & s_{m2} & \ldots & s_{mn} \end{array}\right]$$

where *n* is the number of information sources, that is, the number of sub-feature extraction units.

### 3.3. Feature Fusion Unit

Following the acquisition of multi-modal diagnostic signatures through the heterogeneous sensor feature extraction module, the subsequent integration of cross-domain fault indicators becomes imperative. The proposed hierarchical fusion architecture employs cascaded dense neural layers with nonlinear normalization to achieve parameterized feature consolidation. This framework comprises two principal components: a trainable projection matrix that spatially aligns disparate feature vectors, followed by a probabilistic normalization operator. During fault classification, the latent representations from heterogeneous sensing channels serve as composite inputs to this fusion operator. Through gradient-based optimization, the system autonomously learns optimal weight distributions across sensor modalities corresponding to specific fault patterns. The mathematical formulation of this fusion mechanism can be expressed as:

(5) $$y=wS+b=\left[\begin{array}{c} w^{1}\\ w^{2}\\ \vdots \\ w^{n} \end{array}\right]\left[\begin{array}{cccc} S^{1} & S^{2} & \ldots & S^{n} \end{array}\right]+\left[\begin{array}{c} b^{1}\\ b^{2}\\ \vdots \\ b^{n} \end{array}\right]$$

In this architecture, the predicted value *y* is derived through a fully connected layer that processes input feature vectors *S*, which represent the comprehensive outputs from the multi-source fault feature extraction module. The transformation follows the linear relationship defined by trainable parameters: weight matrix *w* and bias vector *b*. These learnable parameters undergo optimization during training to map the high-dimensional features to classes. Subsequently, the Softmax activation function is applied to convert these classes into normalized probability distributions across predefined fault categories.

### 3.4. Fault Identification Method

The proposed method is to use the multi-information fusion fault recognition network to conduct fusion, which is called complex fusion below. The fusion formula in the network is as follows.

(6) $$O\;\;\;=[O^{S},O^{L}]$$

where *O^S^* = [$o_{1}^{s}$, $o_{2}^{s},\;\ldots ,\;o_{n}^{s}$] is the output of the small convolution kernel network. *O^L^* = [$o_{n+1}^{L}$, $o_{n+2}^{L},\;\ldots ,\;o_{n+m}^{L}$] is the output of the large convolution kernel network. The calculation formula of the full connection layer after splicing layer is as follows.

(7) $$y_{j}=f(\sum_{i=1}^{n}w_{i}o_{i}^{S}+\sum_{i=n+1}^{n+m}w_{i}o_{i}^{L}+b_{j})$$

where *w* and *b* are the weight and bias of the full connection layer. To reduce network parameters and retain effective signal characteristics, a max-pooling function is processed after each small convolution layer as follows.

(8) $$\begin{array}{l} y_{i}^{l}=\max\left(\left\{y_{(i-1)\times N}^{l-1},y_{(i-1)\times N+1}^{l-1},\ldots ,y_{i\times N}^{l-1}\right\}\right),\\ \;\;\;\;\;\;\;\;\;\;\;\;\;\;\;\;\;i\in \left[1,\left\lceil len(y^{l-1})/N\right\rceil \right] \end{array}$$

where *y_i_^l^* is the *i*-th data of the *l* layer, *y^l^*^−1^ is the output of the *l* − 1 layer, *N* is the stride of the pooling, *len* (·) is the length of the vector, ⌈·⌉ is the up rounding, because the padding type is set to same. The corresponding full connection layer is as follows.

(9) $$y_{i}^{l}=w_{i}^{l}y_{i}^{l-1}+b_{j}^{l}$$

where *y_i_^l^* is the output, ${y_{i}^{l}}_{-1}$ is the input of full connection layer, *w^l^* and *b^l^* are the weight and bias of the *l* full connection layer.

(10) $${\hat{y}}_{i}=e^{y_{i}}/\sum_{k=1}^{k=K}e^{y_{k}}$$

Within the VCN model framework, the cross-entropy loss function quantifies the divergence between predicted probabilities and ground truth distributions. Here, *ŷ_i_* ∈ [0, 1] denotes the likelihood of a sample being classified into category i through exponential normalization (*e^y^*), where *y_i* represents the i-th input instance and *K* indicates the total class count. The network architecture *f_ψ_* (*x*) processes input fault signals *x* through learnable parameters *ψ*, generating probability estimates *Ŷ* = *f_ψ_* (*x*). Cross entropy serves as a metric to evaluate the dissimilarity between the true distribution *Y* and predicted distribution *Ŷ*: mathematically, it reflects how closely the model’s output approximates the actual labels, with minimized entropy values indicating optimal alignment between predicted and expected probability patterns. This measurement criterion fundamentally operates by calculating the informational distance between the system’s actual outputs and ideal theoretical expectations.

(11) $$L=H(Y,f_{\psi }(x))=H(Y,\;\hat{Y})=-\sum_{i=1}^{i=K}y_{i}\ln{\hat{y}}_{i}$$

where *y_i_* denotes the model’s predicted probability of the sample being classified into the *i*-th category, with *K* indicating the total number of classes. This differentiable loss function enables gradient-based optimization during neural network training, serving as a fundamental metric to quantify the discrepancy between predicted class probabilities and actual target distributions across multi-class classification tasks.

(12) $$\psi^{*}=\underset{\psi }{\underset{︸}{\arg\min}}H(Y,f_{\psi }(x)),x\in D^{train}$$

The optimal neural network configuration is characterized by parameter set ψ^*^, which represents the mathematically ideal weights minimizing prediction errors for labeled input data points (*x*, *y*). To determine this parameter configuration, modern deep learning systems employ computational differentiation through the back-propagation of error gradients across network layers. Specifically, the partial derivatives of the loss function with respect to each weight are calculated using chain rule differentiation, enabling the subsequent application of gradient-based optimization techniques. These calculated gradients drive systematic weight adjustments through iterative minimization algorithms like stochastic gradient descent or its variants, allowing the network parameters to progressively refine their values toward convergence in multi-dimensional space. This optimization process continues until model performance metrics stabilize within the predefined tolerance thresholds.

(13) $$\psi^{\prime }=\psi -\lambda \times \nabla_{\psi }L$$

where *λ* is the learning rate, ∇*_ψ_ L* is the gradient of *L.* The training process of multi-information fusion network is as follows (Algorithm 1).
**Algorithm 1:** The training process of multi-information fusion network**Input:** Training set $D^{\mathrm{train}}$ = {(*x*_1,_
*y*_1_), (*x*_2,_
*y*_2_), …, (*x*_z,_
*y*_z_)}. Learning rate *λ* **Training Process:** **1. Pre-Training:** **(1)** Randomly initialize the weights and bias in the multi-information fusion network.
**(2)** Divide the training raw samples $D^{train}$ into several sub-datasets C***^train^*|*_n_***(*n* = 1, 2,…, *N*). **(3)** Train the *n*-th sub-ConvNet unit and output the features *S^n^*. **(4)** Regroup the training raw samples $D^{train}$ into a new dataset E***^train^***. **(5)** Train the weight unit and output the weight matrix W. **(6)** Reserve the learned parameters of the sub-ConvNet units and weight unit, and output them as the initial values for the DICN training. **2. Network training:** **(1) Repeat** **(2) For all (*x*_i*,*_
*y*_i_)∈** $D^{train}$ **do**
**(3)**      Input the vibration signal data $D^{train}$ into DICN to calculate the probability of fault type: ${\hat{y}}_{i}=\Gamma_{\Phi }(x_{i})$. **(4)**        Calculate the gradient of parameter ∇_Φ_*L*. **(5)**        Update the weight and bias: $\Phi^{\prime }\leftarrow \Phi -\lambda \times \nabla_{\Phi }L\;.$ **(6)        End** **(7)    Until**    *L* < σ (σ is the set value) or reach the set number of cycles **Output:** Optimal weight Φ_opt_ **Testing Process:** The fault samples x under unknown working condition are input into the trained net-work Γ_Φ*_, and output the optimal classification results Y* = Γ_Φopt_ (x).

## 4. Experimental Validation

### 4.1. Identification Effect Evaluation Method

To verify the superiority of the proposed multi-information fusion network, three methods are used to process the dataset. The first method is to input the data of each measuring point in the dataset directly into the network without fusion, and process it with a single information source, respectively. The second method is to simply stack the data of each information source in the dataset, that is, the one-dimensional vibration time domain signal is simply stacked layer by layer as the input of the network. This method is called simple fusion below. The proposed method is to use the multi-information fusion fault recognition network to complete the fusion, which is called complex fusion below. Secondly, in order to carry out quantitative analysis, the fault recognition accuracy and clustering factor are selected to judge the effect of the information fusion. The accuracy of fault identification is the percentage of the correct number of fault identifications in the total sample. Different information sources have different responses to different types of faults. For the fault data collected by each information source, the between class distance reflects the ability of the information source to distinguish different states, and the within class distance reflects the aggregation of the information source in the same state. Therefore, the larger the between class distance and the smaller the within class distance indicate that the information source has better sensitivity and state discrimination ability. The clustering factor of each information source is calculated as follows. Taking the class *j* fault sample obtained at the *i* th information source as an example, the within class distance of a fault is defined as:

(14) $$d_{i}^{j}=\sum_{m=1}^{N}\sum_{n=1}^{N}\left\|P_{i,j}(m)-P_{i,j}(n)\right\|/\left(N(N-1)\right)$$

where *N* represents the total number of sample data, *P_i_*_,*j*_(⋅) represents the *m*-th sample data of the information source *i* under the *j*-th fault, and ‖⋅‖ represents the Euclidean distance. Therefore, the within class distance of the *i* th information source is calculated as

(15) $$d_{i}=\sum_{j=1}^{M}d_{i}^{j}/M$$

where *M* represents the number of fault categories. Taking the type *j* fault sample obtained at the *i* th information source as an example, the type *j* fault mean is defined as

(16) $$q_{i}^{j}=\sum_{n=1}^{N}P_{i,j}(n)/N$$

Then the between class distance of the *i* th information source is defined as

(17) $$q_{i}=\sum_{j=1}^{M}\sum_{m=1}^{M}\left\|q_{i}^{j}-q_{i}^{m}\right\|/\left(M(M-1)\right)$$

Therefore, the cluster factor of the *i* th information source is defined as

(18) $$\alpha_{i}=q_{i}/d_{i}$$

In summary, this paper uses fault recognition accuracy and cluster factor to judge the effect of fault recognition. The larger the value of these two indicators, the better the effect of fault recognition.

In order to verify the effectiveness of the proposed multi-information fusion fault identification network model for gearbox fault sample identification, this section uses the gearbox dataset published by the Southeast University [31] and the dataset collected by the comprehensive transmission gearbox state signal experiment cooperated by the Chongqing University and the research institute. The SEU Dataset and the CQU Dataset are used to represent the gearbox datasets of the Southeast University and the Chongqing University, respectively.

### 4.2. Case I: Analysis on SEU Dataset

The gearbox dataset is obtained through Spectra Quest’s Drivetrain dynamic simulator (DDS) [31]. The motor provides power for the entire system, which is controlled by the motor controller. The power is transmitted to the planetary gearbox and then to the parallel gearbox. The brake, adjusted by the brake controller, provides a stable load for the gearbox. In this experiment, four common gear faults are designed—broken teeth, missing teeth, root cracks, and tooth surface wear—are combined with normal gears. There are five gear states in this experiment.

**Figure 5:**
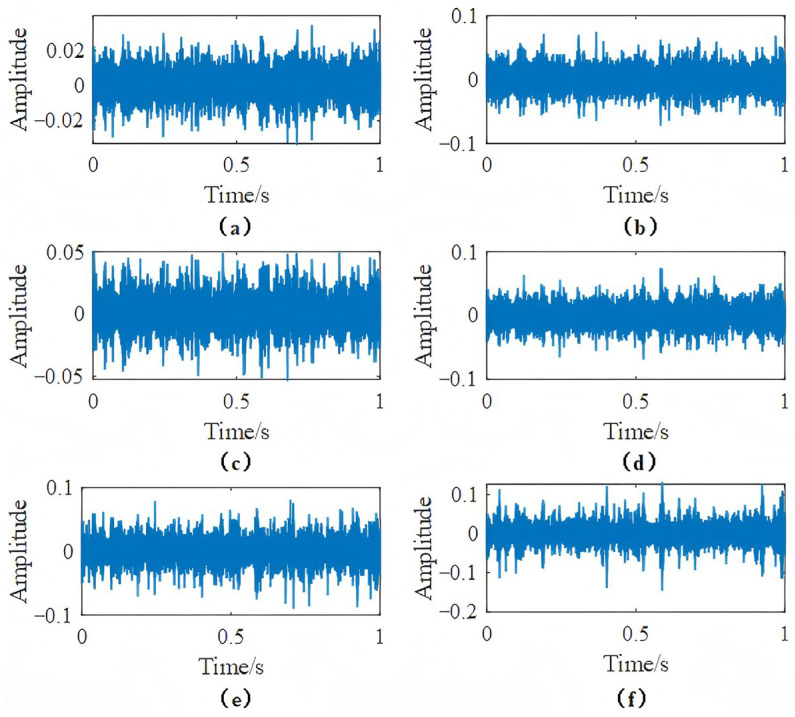
Time domain signal under tooth root crack state of (**a**) measuring point 2, (**b**) measuring point 3, (**c**) measuring point 4, (**d**) measuring point 6, (**e**) measuring point 7, (**f**) measuring point 8.

The whole system is equipped with seven acceleration sensors and a torque sensor. The specific information of each sensor measuring point is shown in Table 2. Since this paper focuses on gearbox components and vibration signals, only the measuring points in the x, y, z directions of the planetary gearbox and the x, y, z directions of the parallel gearbox are selected for analysis and processing. Each measuring point obtains 1020 samples, each sample contains 2048 data points, and the training set and the test set are installed in a ratio of 7:3. The specific gearbox dataset information is shown in Table 3. Taking the original time domain waveform collected by each measuring point in the missing tooth state as an example, the time domain signal of each measuring point is shown in Figure 5.

The model is built using the PyTorch 2.7 deep learning framework and trained using NVIDIA RTX 2080TI. The learning rate of network parameters is 0.001. It can be clearly seen that the response of each measuring point in the missing tooth state has obvious differences in amplitude and shape. It is necessary to extract and further fuse the fault information of each measuring point.

In order to reduce the influence of different networks, both the network model of the single information source and the sub-model of the multi-information fusion fault identification network adopt WDCNN, whose structure is shown in Table 1. The dataset of Table 3 is used to train and test the model. The classification results of each measuring point and fusion are shown in Table 4. It can be clearly seen that the accuracy of each measuring point is below 93% before information fusion, and the recognition accuracy of half of the measuring points is below 80%, and the clustering factor is below 0.7. It can be seen that the recognition rate is low and the difference in each measuring point is large. It can be considered that the recognition effect of a single measuring point is poor. After simple fusion, it can be seen that the recognition rate and clustering factor have been significantly improved, but there are still misidentified samples, indicating that there is still room for improvement. After the multi-information fusion fault recognition network, the recognition rate reaches 100%, the fault recognition ability is significantly improved, and the cluster factor is also increased to 1.204. This indicates that the fault recognition effect after multi-information fusion fault recognition network processing is significantly improved compared with single-point and simple fusion.

**Figure 6:**
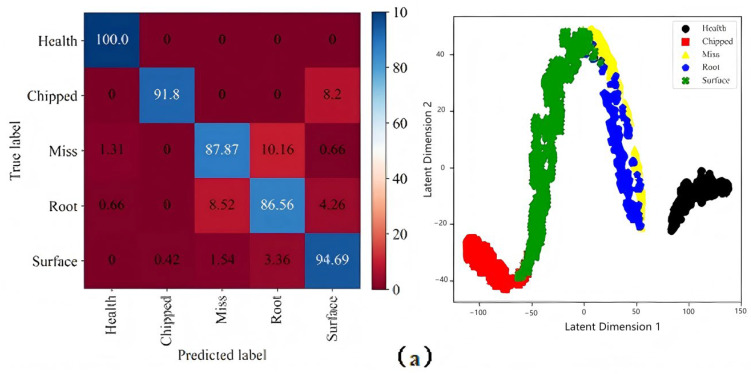

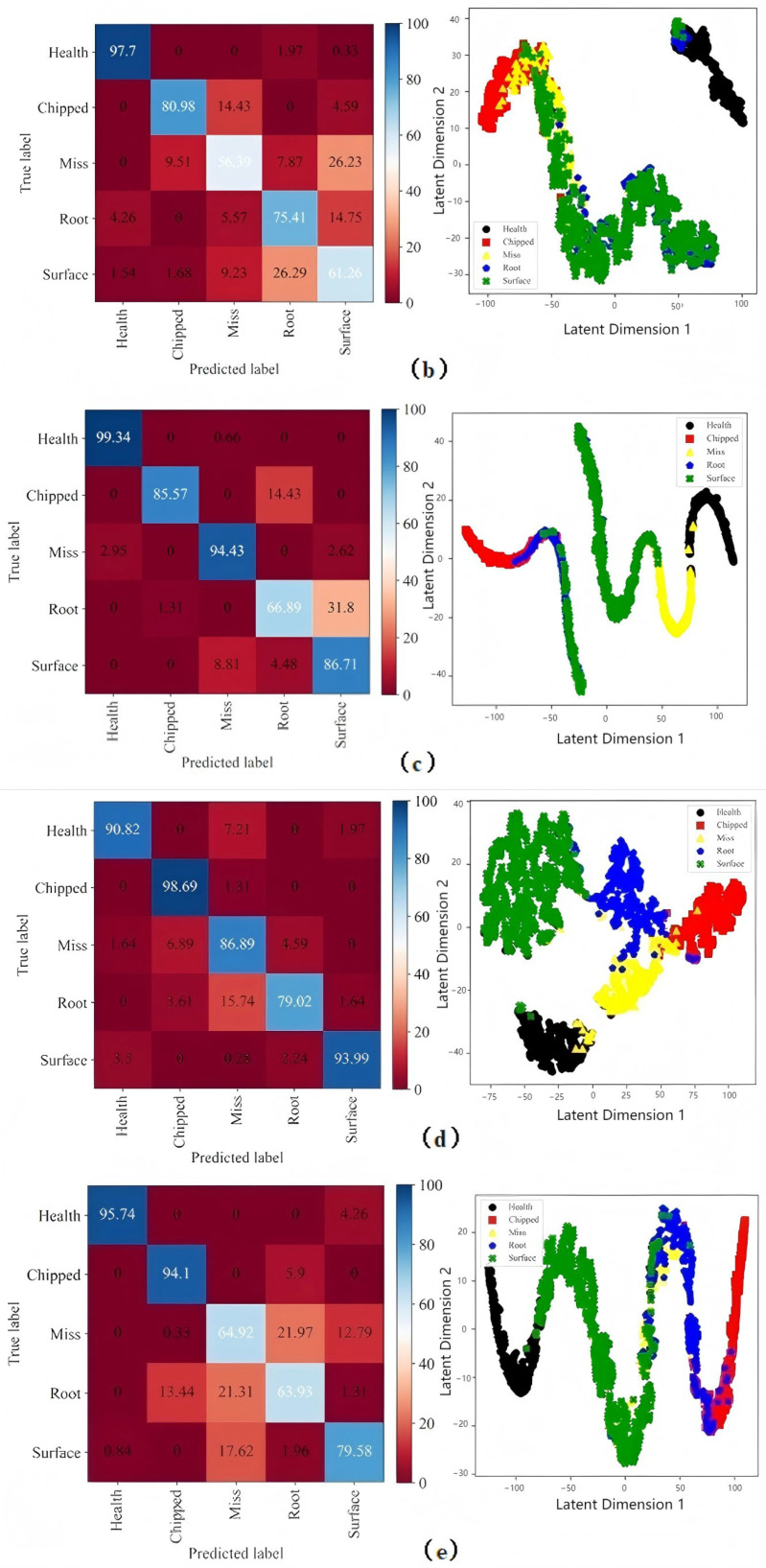

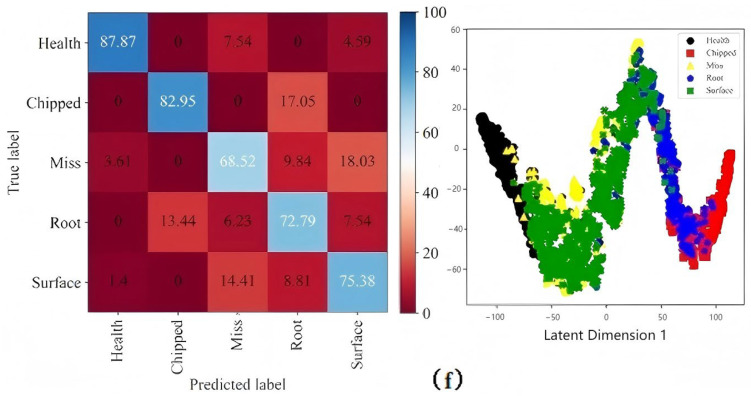
The confusion matrix and cluster graph of each measuring point: (**a**) measuring point 2, (**b**) measuring point 3, (**c**) measuring point 4, (**d**) measuring point 6, (**e**) measuring point 7, (**f**) measuring point 8.

The confusion matrix and cluster graph of each measuring point are shown in Figure 6. For measuring point 2, its recognition of health, tooth root crack, and tooth surface wear state is the highest among all measuring points, but its recognition ability for broken teeth and missing teeth is not strong, and different types of fault samples are mixed together. Each fault category has the problem of a large intra-class distance and a small inter-class distance. For measuring point 3, its recognition accuracy of various states fluctuates greatly, and its recognition rate of missing teeth state is the lowest. Similarly, different types of fault samples are mixed together, for measuring point 4; its recognition accuracy of missing tooth state is the highest among all the measuring points, its recognition effect of tooth root crack state is poor, and it can be seen from its cluster graph that the intra-class distance under the tooth surface wear fault category is large. For measuring point 6, its recognition of broken tooth state is the highest among all the measuring points, and the intra-class and inter-class distances between faults also have the above problems. For measuring points 7 and 8, their identification of various fault states is at an intermediate level among all the measuring points, and the intra-class and inter-class distances between faults also have the above problems. In summary, the same measuring point has different responses to different gear states, and different measuring points have different responses to the same gear state. There is no measuring point that can be fully identified. Each measuring point has its own advantages and disadvantages, and all points have the same fault class. The sample distance is large, and the distance between different fault samples is small.

**Figure 7:**
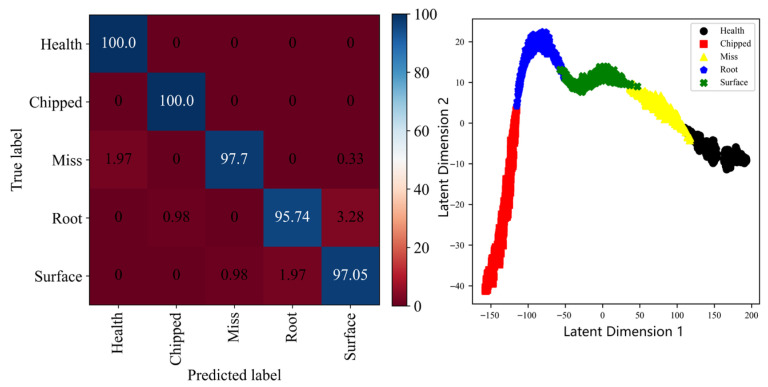
The confusion matrix and cluster graph after simple fusion on SEU Dataset.

In order to make up for the shortcomings of each single measuring point, multiple input signals are simply fused to obtain a confusion matrix and cluster graph as shown in Figure 7. It can be seen that compared with the results of the single measuring point, the recognition accuracy rate has been significantly improved, and the recognition accuracy rate of the broken tooth fault has even reached 100%. However, from its clustering map, it can be seen that all varieties of fault samples are close to each other, and the aliasing phenomenon is more obvious. The results show that compared with a single measurement, the fault recognition effect is improved after simple fusion.

**Figure 8:**
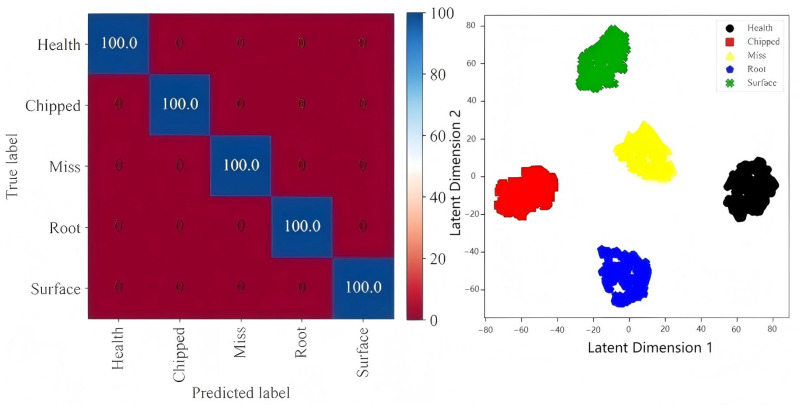
The confusion matrix and cluster graph of the proposed method.

Using multi-information fusion for further analysis, the confusion matrix and cluster graph after multi-information fusion are shown in Figure 8. It can be clearly seen that the recognition accuracy of each fault type after fusion by the proposed method reaches 100%, and from its cluster graph, it can also be seen that the fault distance of the same type is close, and the fault distance of different types is far. This shows that after the information fusion of the multi-information fusion fault identification network, the ability of the neural network to characterize and identify the fault characteristics of the gearbox is improved, and the performance of the multi-information fusion fault identification network model is proved.

To verify the stability and superiority of the model, indicators such as accuracy, recall rate, and F1 score of each fault sample are introduced for evaluation. The expressions for precision, recall, and F1 score are shown in Formulas (19), (20), and (21), respectively.

(19) $$Precision=\frac{TP}{TP+FP}$$

(20) $$Recall=\frac{TP}{TP+FN}$$

(21) $$F1=\frac{2\times Precision\times Recall}{Precision+Recall}$$

where the TP representative is predicted as 1, and is actually 1, then the prediction is correct. Where the FP representative predicted value is 1, but the actual value is 0, this indicates an incorrect prediction. Where the FN representative predicted value is 0, but is actually 1, there is a prediction error. Where the TN representative predicted value is 0, and the actual value is 0, then the prediction is correct.

The recall rates of different faults for each method are shown in Table 5. The first six methods are the single measurement points, while the last two methods are simple fusion and the method proposed in this paper. Through observation, it can be seen that the recall rate of healthy samples is the highest, while the recall rate of tooth root faults using the single measurement point method is the lowest. Most of the root fault samples are incorrectly identified as surface faults. The root fault recall rate of the simple fusion method has been improved, but only by 95.74%. The recall rate of the method proposed in this article is 100%.

The precision and F1 score of different fault samples at each measuring point are shown in Table 6 and Table 7, respectively. The precision and F1 score of simple fusion are significantly higher than those of the single measurement points, and the method proposed in this paper has an evaluation score which is about 3% higher than that of simple fusion. This indirectly validates the superiority of the proposed method.

### 4.3. Case II: Analysis on CQU Dataset

**Figure 9:**
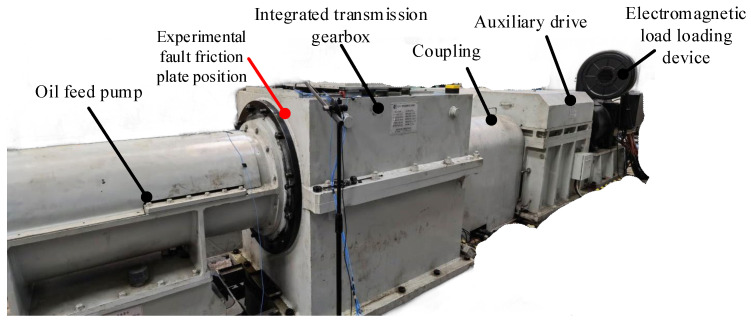
Gearbox fault test bench.

The overall composition of the test bench is shown in Figure 9, and the oil supply pump provides power for the entire transmission system. The integrated transmission gearbox is the core component, which is equipped with gears and sensors, and a replaceable tooth friction plate is installed inside to simulate the typical faults of the friction plate. The coupling is used to connect the rear load device, the auxiliary transmission device is used to connect the electromagnetic load loading device and the front components, and the electromagnetic load loading device is used to provide a freely adjustable load.

**Figure 10:**
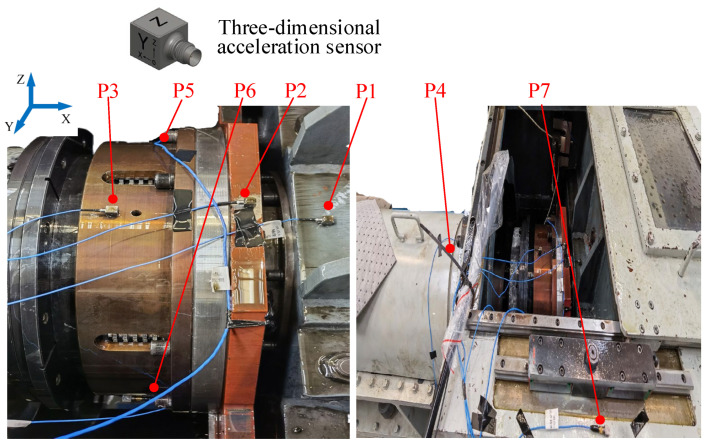
Sensor layout diagram.

In order to obtain comprehensive state information during the operation of mechanical equipment, multiple measuring points need to be arranged at different locations. In order to verify the proposed multi-information fusion method, a dense measurement point scheme is proposed, and sensors are arranged in as many locations as possible. The fault is set on the friction plate of the gearbox, and the sensor is arranged near the friction plate. Therefore, in order to obtain the response information from the friction plate in different states, a three-way vibration acceleration sensor is arranged at seven positions according to Figure 10, where sensor 1 is located on the bearing base, sensor 2 is located on the rocker arm, sensor 3 is located on the upper side of the outer hub, sensor 4 is located on the outer side of the shell, sensor 5 is located on the left side of the outer hub, sensor 6 is located on the right side of the outer hub, and sensor 7 is located on the test bench box.

**Figure 11:**
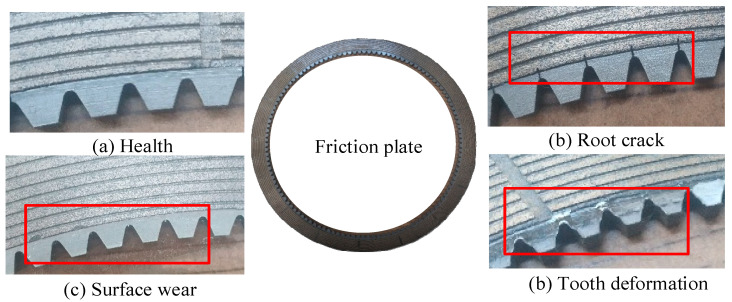
Friction plate fault physical diagram.

Following common fault types of gearbox friction plate, the root crack, tooth surface wear, and tooth deformation of friction plate are selected in the experiment. Combined with the friction plate in normal state, there are four types of friction plate states. The fault is set on the tooth friction plate of the integrated transmission gearbox. The fault of the tooth friction plate is shown in Figure 11. The sampling frequency is set to 25 kHz, the sampling time of each group of data is 120 s, and the speed is 1500 rpm.

In order to facilitate the processing of neural networks, a multi-information source friction plate gear fault sample dataset is constructed based on the collected data. In this experiment, in each friction plate state, 3050 samples are obtained for each measuring point by a sliding window. Each sample contains 2048 data points. The training set and test set are divided according to the ratio of 7:3. The specific friction plate multi-source dataset information is shown in Table 8.

**Figure 12:**
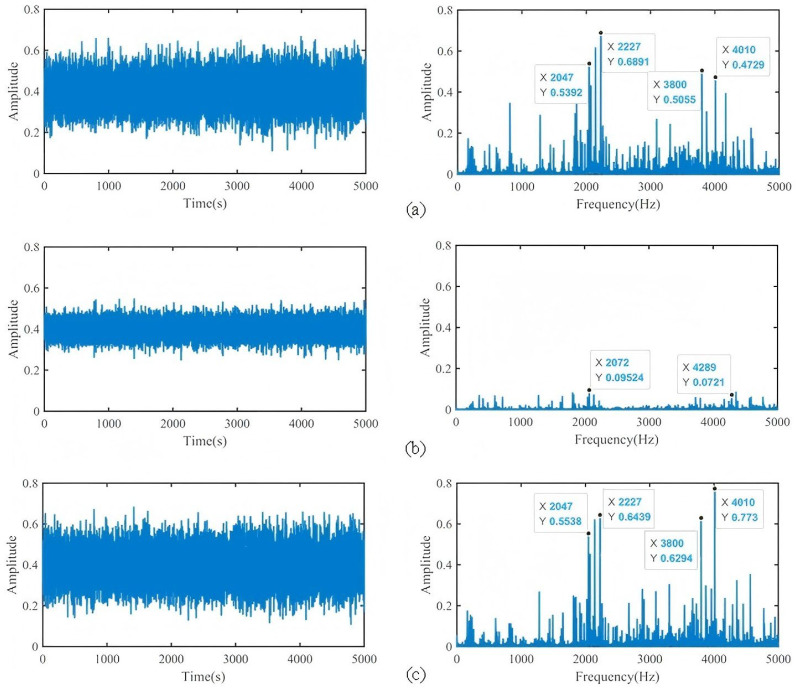
Time domain and frequency domain diagrams of y-direction signals of different sensors: (**a**) P1 y-direction, (**b**) P2 y-direction, (**c**) P3 y-direction.

Taking the P1-y direction of the bearing seat, the P2-y direction of the rocker arm, and the P3-y direction of the outer hub as an example, the vibration signals collected under the condition of the tooth root crack are analyzed. The results are shown in Figure 12.

It can be seen from the time domain that the signal amplitude level collected at P1-y and P3-y is significantly greater than that of P2-y. The signal is divided into 10 segments on average, and the average peak-to-peak value is counted. P1-y and P3-y are about 8, while P2-y is only 4.78. From the spectrum point of view, the maximum amplitude at P1-y and P3-y is also greater than that in the P2-y direction. Among them, compared with the spectrum structure of P1-y and P3-y, the frequency components contained are more consistent, and the sidebands are uniform and obvious at 2000 Hz and 4000 Hz, while the spectrum information of P2-y is not as rich as the other two positions. Therefore, the above conclusions show that the P1-y direction of the bearing seat and the P3-y direction of the outer hub are more sensitive to the fault information, and contain similar information. The P2-y direction of the rocker arm contains less information and has a weak response to the fault. This shows that the information intensity and fault information reflected by the vibration data collected by the gearbox at different positions are different, the information sensitivity is different, and the fault response intensity is different. Therefore, it is necessary to extract and fuse the fault information of the multi-point vibration data of the gearbox.

**Figure 13:**
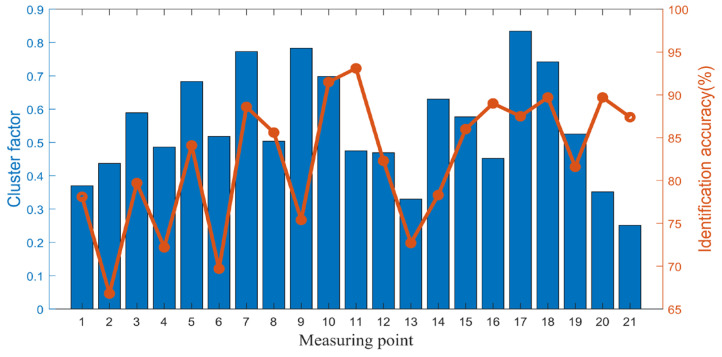
The identification results of each measuring point.

Firstly, the fault identification of each sensor measuring point is carried out separately, and the results are shown in Table 9 and Figure 13. It can be clearly seen that the accuracy of each measuring point is below 93% and the clustering factor is below 0.8 before information fusion. Due to the large number of measuring points, in order to reflect the superiority of the multi-information fusion network, only the first three measuring points with a high fault recognition rate, namely measuring points 10, 11, and 20, are selected for subsequent comparative analysis.

**Figure 14:**
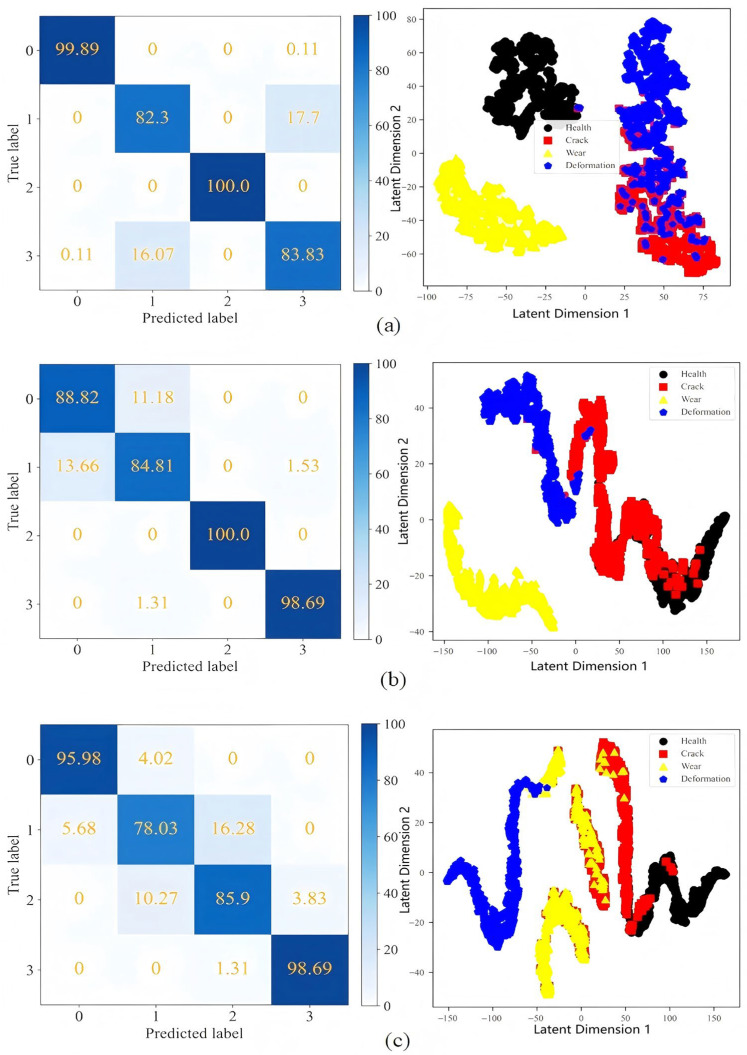
The confusion matrix and cluster graph of measuring points 10, 11, and 20. (**a**) measuring point 10, (**b**) measuring point 11, (**c**) measuring point 20.

The fault identification results of measuring points 10, 11, and 20 are visualized, and the confusion matrix and cluster graph of each measuring point are obtained as shown in Figure 14. For measuring point 10, its recognition accuracy for wear and health status is the highest, but its recognition rate for other fault states is low, below 85%. Its cluster graph reflects that the distance between various fault samples of the measuring point is close, and there is aliasing phenomenon. In particular, the deformation and crack state samples overlap more, so the measuring point has a better recognition effect on wear and health status, and a worse recognition effect on deformation and crack state. Similarly, it can be seen that measuring point 11 has a good recognition effect on wear and deformation state, and a poor recognition effect on health and crack state. Measuring point 20 has a good recognition of health and deformation state, and a poor recognition of crack and wear state.

Next, the simple fusion method and the proposed method are used to further analyze measuring points 10, 11, and 20, respectively. The results are shown in Table 10. After simple fusion, the recognition rate is not lower than that of a single measuring point, but it is not significantly improved. The clustering factor is higher than that of a single measuring point before fusion, indicating that simple fusion cannot effectively fuse information and needs further improvement. After the fusion of the proposed method, the fault recognition rate reaches 99.9%, and the fault recognition ability is significantly improved, which is higher than that of all single measuring points. The clustering factor is also increased to 0.772, which is higher than that of single measuring point and simple fusion. Therefore, it shows that after multi-information fusion fault identification network processing, the fault identification ability is significantly improved compared with a single measurement point and simple fusion.

**Figure 15:**
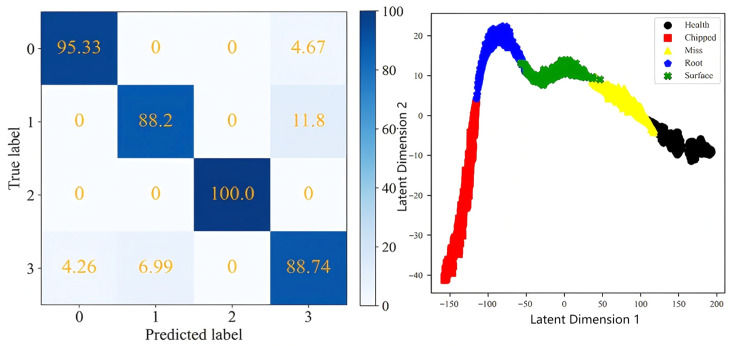
The confusion matrix and cluster graph after simple fusion on CQU Datase.

**Figure 16:**
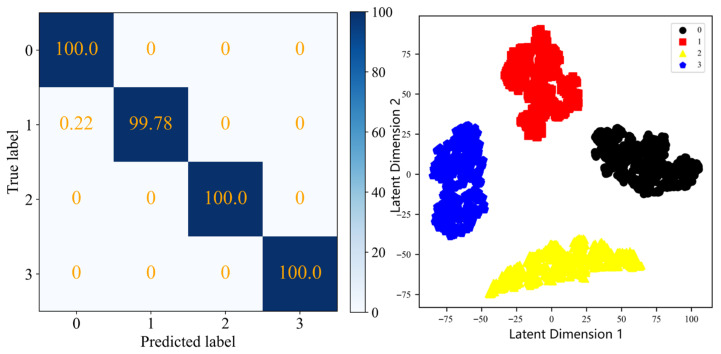
The confusion matrix and cluster graph after fusion using the proposed method.

The results obtained by the simple fusion method and the proposed method are visualized, and the confusion matrix and cluster graph are shown in Figure 15 and Figure 16. For simple fusion, it can be seen that compared with the results of a single measuring point, the recognition accuracy is not significantly improved, and its cluster graph also reflects the aliasing of various fault samples, indicating that the fault recognition effect after simple fusion is not ideal, and the information cannot be fully and effectively fused for fault recognition. After the fusion of the proposed multi-information fusion fault identification network, the recognition accuracy of each fault type reaches more than 99.9%. The cluster graph also reflects that the similar fault samples have a high degree of aggregation, and different types of fault samples are also far apart, with good distinguishability. This shows that the multi-information fusion fault identification network has a better representation and recognition ability for gear fault characteristics, and proves the superiority of the multi-information fusion fault identification network model.

**Figure 17:**
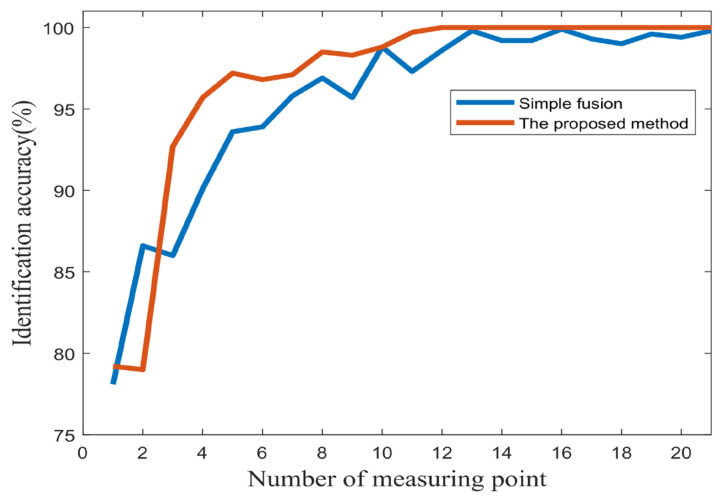
The change curve of fault recognition accuracy with the number of measuring points.

Furthermore, in order to explore the difference in recognition accuracy between the simple fusion method and the proposed method under a different number of measuring points, the number of fused measuring points is increased from 1 to 21 to consider the change in recognition accuracy. The results are shown in Figure 17. It can be clearly seen that with the increase of the number of measuring points, the recognition accuracy of the proposed method is basically higher than that of simple fusion, and with the increase of the number of measuring points, the proposed method has better monotonicity, and the recognition accuracy will not decrease with the increased amount of information. The recognition accuracy of simple fusion fluctuates with the increase of the number of measuring points, which shows that when the fused information has relatively poor signals, the proposed method is more resistant to its adverse effects than simple fusion, which proves that the multi-information fusion fault recognition network performs better than simple information fusion.

The recall rates of different faults for each method are shown in Table 11. The first six methods are the single measurement points, while the last two methods are simple fusion and the method proposed in this paper. Through observation, it can be seen that the recall rate of healthy samples is the highest, while the recall rate of tooth root faults using the single measurement point method is the lowest. Most of the root fault samples are incorrectly identified as surface faults. The root fault recall rate of the simple fusion method has been improved, but only by 88.20%. The recall rate of the method proposed in this article is 99.78%.

The precision and F1 score of different fault samples at each measuring point are shown in Table 12 and Table 13, respectively. The precision and F1 score of simple fusion are significantly higher than those of the single measurement points, and the method proposed in this paper has an evaluation score which is about 6% higher than that of simple fusion. This indirectly validates the superiority of the proposed method.

## 5. Conclusions

On the basis of a convolutional neural network, this paper extends the concept of ensemble learning to information fusion, and uses the relevant methods of ensemble learning to carry out information fusion. Therefore, a multi-information fusion fault recognition network model based on deep ensemble learning is designed. It is composed of a multi-information source fault feature extraction unit, a feature fusion unit, and an output layer, which solves the problem of an uneven recognition effect and a high misdiagnosis rate of single information source in the field of mechanical fault recognition. The performance of the multi-information fusion deep neural network fault recognition model is verified and analyzed on the Southeast University gearbox dataset and the Chongqing University friction plate gearbox dataset. The proposed method is compared with the single measurement point without fusion and simple stacking fusion. The results show that after the fusion of the proposed network model, the recognition accuracy of the fault types is significantly improved, close to 100%, and the intra-class distance of each fault type sample is reduced, the inter-class distance becomes larger, and the stability is improved. In addition, the method proposed in this article does not consider the situation where sensor failures or other reasons lead to data loss, which is also the direction and focus of our future research.

## Figures and Tables

**Table 1:** Architecture of WDCNN.

Network Layer	Model Parameters	Output Size (Width × Depth)
Convolution 1	Conv1 (16@1 × 64, Stride = 16)	128 × 16
Pooling 1	MaxPooling1 (1 × 2, Stride = 2)	64 × 16
Convolution 2	Conv2 (16@1 × 3, Stride = 1)	64 × 32
Pooling 2	MaxPooling2 (1 × 2, Stride = 2)	32 × 32
Convolution 3	Conv3 (32@1 × 3, Stride = 1)	32 × 64
Pooling 3	MaxPooling3 (1 × 2, Stride = 2)	16 × 64
Convolution 4	Conv4 (64@1 × 3, Stride = 1)	16 × 64
Pooling 4	MaxPooling4 (1 × 2, Stride = 2)	8 × 64
Convolution 5	Conv5 (64@1 × 3, Stride = 16)	6 × 64
Pooling 5	MaxPooling5 (1 × 2, Stride = 2)	3 × 64
Fully connected	100	100 × 1
Softmax	5	5

**Table 2:** Measuring point information table on the SEU dataset.

Measuring Point	Sensor Type	Location
1	Acceleration	Motor
2	Acceleration	Planetary gearbox (x direction)
3	Acceleration	Planetary gearbox (y direction)
4	Acceleration	Planetary gearbox (z direction)
5	Torque	Between motor and planetary gearbox
6	Acceleration	Parallel gearbox (x direction)
7	Acceleration	Parallel gearbox (y direction)
8	Acceleration	Parallel gearbox (z direction)

**Table 3:** The SEU dataset contains information on different fault categories.

Measuring Point	Fault Type	Label	Data Length	Number of Train/Test Samples
Measuring point 2/3/4/6/7/8	Health	0	2048	715/305
	Chipped	1	2048	715/305
	Miss	2	2048	715/305
	Root	3	2048	715/305
	Surface	4	2048	715/305

**Table 4:** The results of different methods on the SEU dataset.

Measuring Point	Average Accuracy (%)	Cluster Factor
Measuring point 2	92.7	0.666
Measuring point 3	71.6	0.520
Measuring point 4	86.6	0.485
Measuring point 6	90.7	0.514
Measuring point 7	79.6	0.285
Measuring point 8	77.1	0.296
Simple fusion	98.1	1.007
The proposed method	**100**	**1.204**

**Table 5:** Recall rate of different faults at the measuring points on SEU Dataset.

Measuring Point	Health	Chipped	Miss	Root	Surface
Point 2	100%	91.80%	87.87%	86.56%	94.69%
Point 3	97.70%	80.98%	56.39%	75.41%	61.26%
Point 4	99.34%	85.57%	94.43%	66.89%	86.71%
Point 6	90.82%	98.69%	86.89%	79.02%	93.99%
Point 7	95.74%	94.10%	64.92%	63.93%	79.58%
Point 8	87.87%	82.95%	68.52%	72.79%	75.38%
Simple fusion	100%	100%	97.7%	95.74%	97.05%
Proposed method	100%	100%	100%	100%	100%

**Table 6:** The precision of different faults at the measuring points on SEU Dataset.

Measuring Point	Health	Chipped	Miss	Root	Surface
Point 2	98.07%	99.54%	89.73%	86.49%	87.83%
Point 3	94.40%	87.86%	65.86%	67.61%	57.17%
Point 4	97.12%	98.49%	90.89%	77.96%	71.58%
Point 6	94.63%	90.38%	77.98%	92.04%	96.30%
Point 7	99.13%	87.23%	62.51%	68.18%	81.25%
Point 8	94.61%	86.01%	70.86%	67.09%	71.42%
Simple fusion	98.07%	99.03%	99.01%	97.98%	96.41%
Proposed method	100%	100%	100%	100%	100%

**Table 7:** The F1-score of different faults at the measuring points on SEU Dataset.

Measuring Point	Health	Chipped	Miss	Root	Surface
Point 2	99.03%	95.51%	88.79%	86.52%	91.13%
Point 3	96.02%	84.28%	60.76%	71.30%	59.14%
Point 4	98.22%	91.58%	92.63%	72.00%	78.42%
Point 6	92.69%	94.35%	82.19%	85.03%	95.13%
Point 7	97.41%	90.53%	63.69%	65.99%	80.41%
Point 8	91.12%	84.45%	69.67%	69.82%	73.35%
Simple fusion	99.03%	99.51%	98.35%	96.85%	96.73%
Proposed method	100.00%	100.00%	100.00%	100.00%	100.00%

**Table 8:** Friction disc fault dataset information table.

Measuring Point	Fault Type	Label	Data Length	Number of Train/Test Samples
Measuring point 1~21	Health	0	2048	2135/915
	Root crack	1	2048	2135/915
	Tooth deformation	2	2048	2135/915
	Surface wear	3	2048	2135/915

**Table 9:** The results of each single measuring point on the CQU dataset.

Measuring Point	Average Accuracy (%)	Cluster Factor
1 (P1-x direction)	78.1	0.370
2 (P1-y direction)	66.8	0.437
3 (P1-z direction)	79.7	0.589
4 (P2-x direction)	72.2	0.486
5 (P2-y direction)	84.1	0.683
6 (P2-z direction)	69.7	0.518
7 (P3-x direction)	88.6	0.773
8 (P3-y direction)	85.6	0.504
9 (P3-z direction)	75.4	0.783
10 (P4-x direction)	91.5	0.698
11 (P4-y direction)	93.1	0.475
12 (P4-z direction)	82.3	0.469
13 (P5-x direction)	72.7	0.330
14 (P5-y direction)	78.3	0.630
15 (P5-z direction)	86.0	0.577
16 (P6-x direction)	89.0	0.452
17 (P6-y direction)	87.5	0.834
18 (P6-z direction)	89.7	0.742
19 (P7-x direction)	81.6	0.525
20 (P7-y direction)	89.7	0.352
21 (P7-z direction)	87.4	0.251

**Table 10:** The results of different methods on the CQU dataset.

Measuring Point	Average Accuracy (%)	Cluster Factor
Measuring point 10	91.5	0.698
Measuring point 11	93.1	0.475
Measuring point 20	89.7	0.352
Simple fusion	93.1	0.739
The proposed method	99.9	**0.772**

**Table 11:** Recall rate of different faults at the measuring points on CQU Datase.

Measuring Point	Health	Root Crack	Tooth Deformation	Surface Wear
Point 10	99.89%	82.30%	100%	83.83%
Point 11	88.82%	84.81%	100%	98.69%
Point 20	95.98%	78.03%	85.90%	98.69%
Simple fusion	95.33%	88.20%	100%	88.74%
Proposed method	100%	99.78%	100%	100%

**Table 12:** The precision of different faults at the measuring points on CQU Datase.

Measuring Point	Health	Root Crack	Tooth Deformation	Surface Wear
Point 10	99.89%	83.66%	100%	82.48%
Point 11	86.67%	87.16%	100%	98.47%
Point 20	94.41%	84.52%	83.00%	96.26%
Simple fusion	95.33%	92.66%	100%	84.35%
Proposed method	99.78%	100%	100%	100%

**Table 13:** The F1-score of different faults at the measuring points on CQU Datase.

Measuring Point	Health	Root Crack	Tooth Deformation	Surface Wear
Point 10	99.89%	82.97%	100.00%	83.15%
Point 11	87.73%	85.97%	100.00%	98.58%
Point 20	95.19%	81.15%	84.43%	97.46%
Simple fusion	95.33%	90.38%	100.00%	86.49%
Proposed method	99.89%	99.89%	100.00%	100.00%
